# Long-Term Results After Senning and Mustard Operations for d-Transposition of the Great Arteries: Atrial Switch Should Remain in Armamentarium

**DOI:** 10.1093/ejcts/ezag126

**Published:** 2026-03-21

**Authors:** Fabian A Kari, Emre Ortac, Sebastian G Michel, Julie Cleuziou, Paul P Heinisch, Zahra Alalawi, Fabian von Scheidt, Doris Kienmoser, Masamichi Ono, Harald Kämmerer, Hans Meisner, Rüdiger Lange, Jürgen Hörer

**Affiliations:** Department of Pediatric and Congenital Cardiac Surgery, TUM University Hospital German Heart Center, Munich, 80636, Germany; Department of Cardiac Surgery, Section of Pediatric and Congenital Cardiac Surgery, LMU University Hospital, Munich 81377, Germany; European Pediatric Heart Center EKHZ Munich, Munich, Germany; Department of Pediatric and Congenital Cardiac Surgery, TUM University Hospital German Heart Center, Munich, 80636, Germany; Department of Cardiac Surgery, Section of Pediatric and Congenital Cardiac Surgery, LMU University Hospital, Munich 81377, Germany; European Pediatric Heart Center EKHZ Munich, Munich, Germany; Department of Pediatric and Congenital Cardiac Surgery, TUM University Hospital German Heart Center, Munich, 80636, Germany; Department of Cardiac Surgery, Section of Pediatric and Congenital Cardiac Surgery, LMU University Hospital, Munich 81377, Germany; European Pediatric Heart Center EKHZ Munich, Munich, Germany; Department of Pediatric and Congenital Cardiac Surgery, TUM University Hospital German Heart Center, Munich, 80636, Germany; Department of Cardiac Surgery, Section of Pediatric and Congenital Cardiac Surgery, LMU University Hospital, Munich 81377, Germany; European Pediatric Heart Center EKHZ Munich, Munich, Germany; Department of Pediatric and Congenital Cardiac Surgery, TUM University Hospital German Heart Center, Munich, 80636, Germany; Department of Cardiac Surgery, Section of Pediatric and Congenital Cardiac Surgery, LMU University Hospital, Munich 81377, Germany; European Pediatric Heart Center EKHZ Munich, Munich, Germany; Department of Pediatric and Congenital Cardiac Surgery, TUM University Hospital German Heart Center, Munich, 80636, Germany; Department of Cardiac Surgery, Section of Pediatric and Congenital Cardiac Surgery, LMU University Hospital, Munich 81377, Germany; European Pediatric Heart Center EKHZ Munich, Munich, Germany; Department of Congenital Heart Defects and Pediatric Cardiology, TUM University Hospital German Heart Center, Munich, Germany; Department of Pediatric and Congenital Cardiac Surgery, TUM University Hospital German Heart Center, Munich, 80636, Germany; Department of Pediatric and Congenital Cardiac Surgery, TUM University Hospital German Heart Center, Munich, 80636, Germany; Department of Cardiac Surgery, Section of Pediatric and Congenital Cardiac Surgery, LMU University Hospital, Munich 81377, Germany; European Pediatric Heart Center EKHZ Munich, Munich, Germany; Department of Congenital Heart Defects and Pediatric Cardiology, TUM University Hospital German Heart Center, Munich, Germany; Department of Pediatric and Congenital Cardiac Surgery, TUM University Hospital German Heart Center, Munich, 80636, Germany; Department of Cardiac Surgery, Section of Pediatric and Congenital Cardiac Surgery, LMU University Hospital, Munich 81377, Germany; European Pediatric Heart Center EKHZ Munich, Munich, Germany; Department of Pediatric and Congenital Cardiac Surgery, TUM University Hospital German Heart Center, Munich, 80636, Germany; Department of Cardiac Surgery, Section of Pediatric and Congenital Cardiac Surgery, LMU University Hospital, Munich 81377, Germany; European Pediatric Heart Center EKHZ Munich, Munich, Germany; Department of Pediatric and Congenital Cardiac Surgery, TUM University Hospital German Heart Center, Munich, 80636, Germany; Department of Cardiac Surgery, Section of Pediatric and Congenital Cardiac Surgery, LMU University Hospital, Munich 81377, Germany; European Pediatric Heart Center EKHZ Munich, Munich, Germany

**Keywords:** transposition of great arteries, atrial switch operation, d-transposition, systemic right ventricle

## Abstract

**Objectives:**

The Senning and Mustard atrial switch procedures, once the standard surgical treatment for transposition of the great arteries, carry the long-term risks linked to a systemic right ventricle.

**Methods:**

A retrospective follow-up study was conducted to characterize long-term outcomes focusing on survival, baffle-related, and non-baffle-related reoperations.

**Results:**

*N* = 417 patients with d-transposition of the great arteries (d-TGA) (70% male) were treated with an atrial switch operation in one centre (*n* = 88 Mustard, *n* = 329 Senning, treated in 1974-2001). The mean follow-up time was 29.7 (SD 14.2) years, with a median follow-up of 34.2 years (range 10-50 years). The average age of the patients at the last follow-up was 30.9 (SD 14.1) years. Overall survival at 40 years was 78.3% (95% CI, 74.0%-82.8%) with *n* = 128 patients under observation. Forty-year survival was 81.6% (95% CI, 77.0%-86.5%, *n* = 91 at risk) after Senning and 66.7% (95% CI, 57.2%-77.8%, *n* = 37 at risk) after Mustard (log-rank *P* < .001). Freedom from reoperation at 40 years was 73.1% (95% CI, 68.4%-78.0%). After the Senning, long-term survival over 4 decades was slightly inferior to survival of a general population sample.

**Conclusions:**

Given a survival up to the fifth life decade only slightly inferior to a normal population and superiority of the Senning over the Mustard operation, the Senning should remain in the congenital cardiac surgeons’ armamentarium for late presentation of TGA, ccTGA, and other indications.

**Clinical registration number:**

The study is designed as a retrospective study.

## Graphical Abstract

**Figure:**
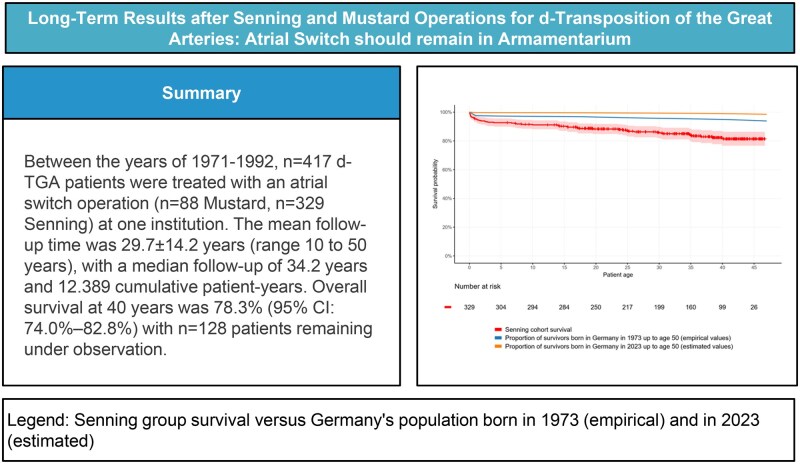

## Introduction

d-Transposition of the great arteries (TGAs) is one of the most common cyanotic heart defects in infancy.^1^ In the mid-1950s, initial attempts at an arterial switch operation were unsuccessful. Patients with TGA were managed using atrial switch procedures, such as the Senning operation (introduced in 1957)^2^ or the Mustard operation (introduced in 1963).^3^ For over 2 decades, these atrial switch operations were the standard surgical treatment for d-TGA.

In 1975, Jatene et al^4^ reported the first successful arterial switch operation. The transition was driven by concerns over long-term systemic right ventricular dysfunction after atrial switch, as well as the increasing incidence of complications related to the surgically created venous pathways.

In this study, long-term survival and freedom from reoperation of patients treated with the Senning or Mustard procedures are presented.

## Patients and methods

### Patient selection and data acquisition

**Figure 1.**
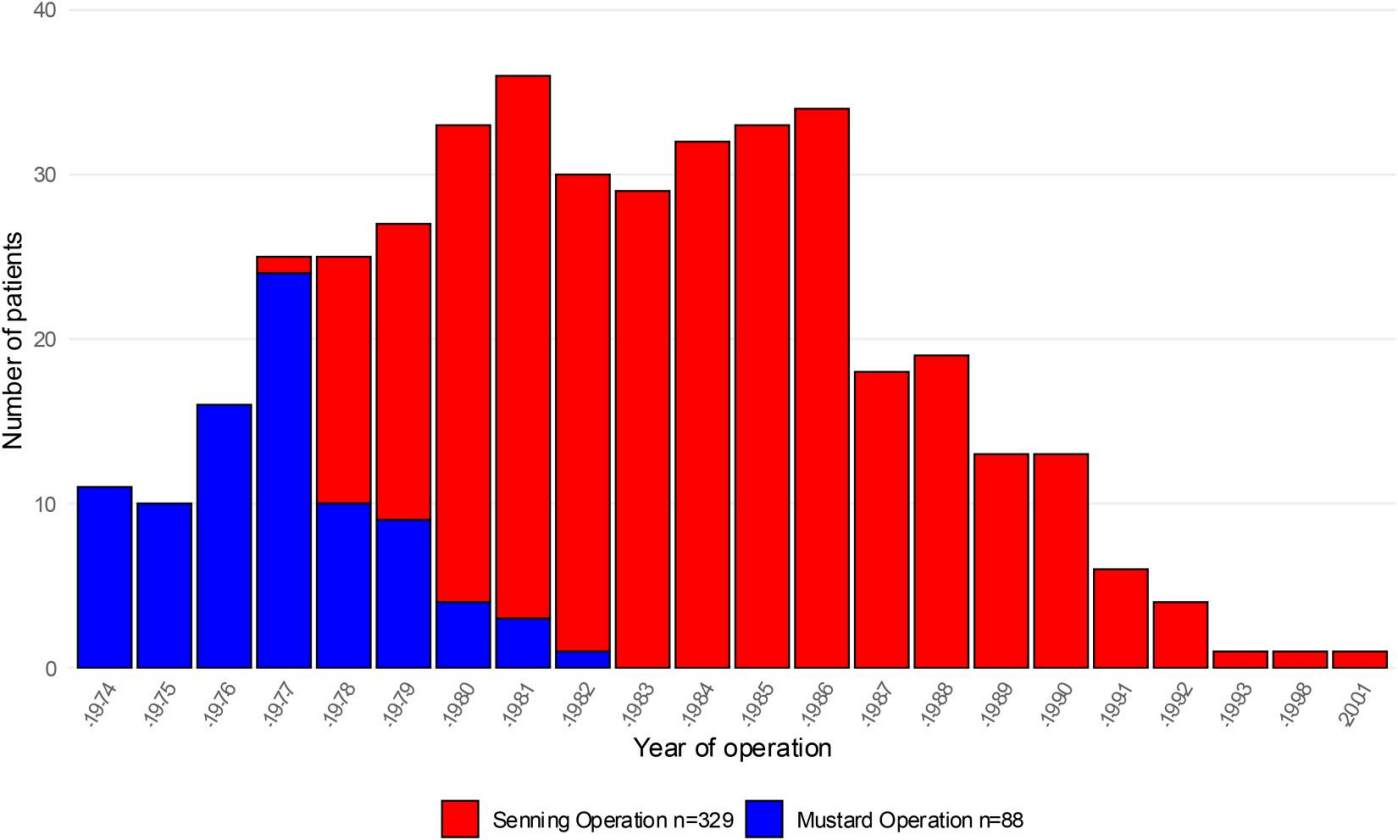
Number of Atrial Switch Operations Per Year by Surgery Type.

All patients who had undergone correction of TGA at the German Heart Center, Technical University of Munich, by means of an atrial switch operation between 1974 and 2001 (last case performed) were included. Between 1974 and 1982, *n* = 88 patients underwent the Mustard operation, and *n* = 329 patients received a Senning operation between 1977 and 2001 (**Figure 1**). The study was conducted according to the Declaration of Helsinki, and it was approved by the Ethics Committee of the Technical University of Munich (2024-41-S-NP).

Informed consent was obtained by sending a written consent form to each patient or their parents, which was signed and returned. To ensure completeness, the medical practitioner was subsequently contacted for follow-up data. Patients who had relocated within Germany could generally be traced through the national address registry, whereas those who had moved abroad (or, if they had lived outside Germany already, had relocated to their country of residence) could not be reached in all cases. The surgical techniques are described in a different publication.^5^

### Data collection and follow-up

**Figure 2.**
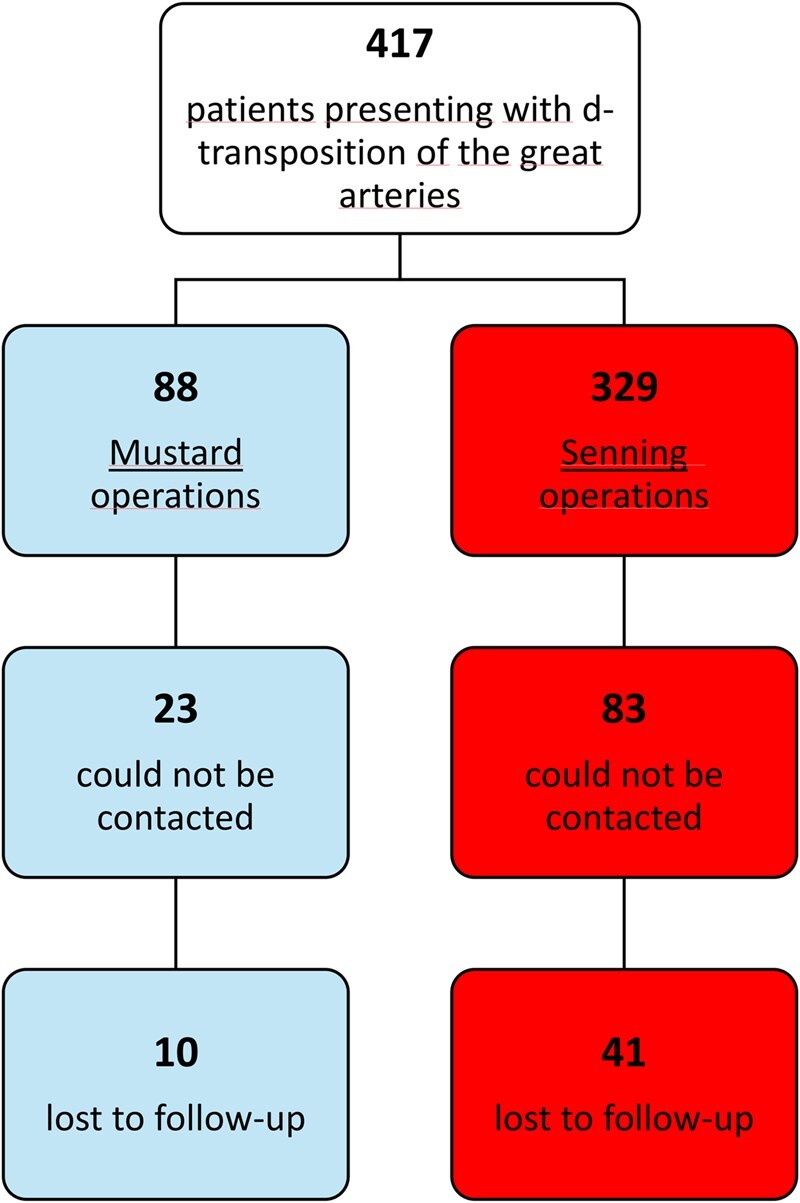
Flowchart of Patients Lost to Follow-Up After Hospital Discharge.

Preoperative, perioperative, and follow-up data were retrospectively analysed with a focus on survival and cardiac reoperations. The updated, mean follow-up duration was 29.7 (SD 14.2) years, with a median of 34.2 years (range, 10-50 years). A total of *n* = 107 patients had attended a clinical visit or had been hospitalized at the German Heart Center within the past 10 years, allowing the study centre to obtain follow-up reports from treating physicians. In addition, *n* = 310 patients were contacted directly between August 2024 and January 2025, of whom *n* = 106 could not be reached. Fifty-one patients had no available follow-up information after hospital discharge and were therefore considered lost to follow-up (**Figure 2**). The overall completeness of follow-up was 88% corresponding to 12 389 cumulative patient-years. Patients whose practitioners or families were unresponsive, and who had died, were identified through the German National Registry. While the status of these patients could be clarified in all cases, the exact cause of death could not always be identified.

### Data analysis and definitions of reoperation types

Descriptive data for continuous variables are presented as means and standard deviation; categorical variables are presented as relative frequencies. In cases of wide variability, the range was reported instead of the standard deviation. The study end-points were defined as death, reoperation for baffle complications, reoperation for right ventricular dysfunction, or reoperation for other indications. Reoperation for baffle complications was defined either as an operation including enlargement of one of the venous pathways, and/or closure of baffle leaks. Reoperation for systemic ventricular failure was defined as: tricuspid valve repair or replacement, pulmonary artery banding (PAB), arterial switch operation including Senning/Mustard take-down, heart transplantation, and mechanical circulatory support (MCS). The probability of freedom from events was estimated according to the Kaplan-Meier method. Freedom-from-event curves were compared using the log-rank test for determining p values. Analyses were performed with RStudio with “survival” package version 3.8-3, and the “survminer” package version 0.5.0.

## Results

### Survival

**Figure 3.**
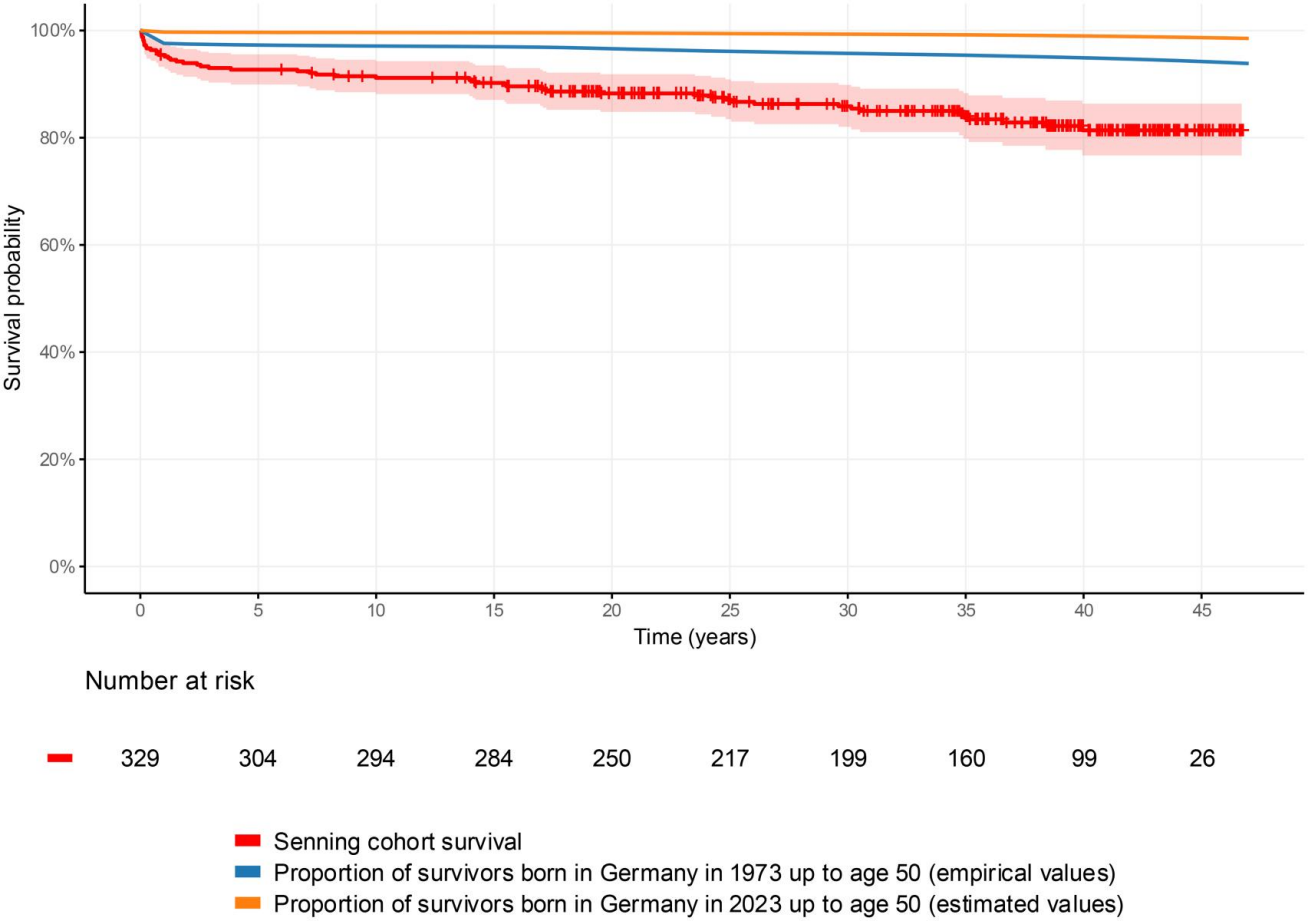
Senning Group Survival Versus Germany’s Population Born in 1973 (Empirical Data) and in 2023 (Estimated Data). Of note, the survival curve for patients after Senning operation includes in-hospital-losses. General population sample curves do not represent Kaplan-Meier calculations, but proportions of survivors at a given year. Note the neonatal losses in the general population sample from 1973.

The overall survival at 40 years of follow-up was 78.3% (95% CI, 74.0%-82.8%) with *n* = 128 patients under surveillance at this time point. The actuarial survival rate of the Senning cohort is shown alongside survival proportions of the general German population born in 1973, when atrial switch procedures were first introduced, and of those born in 2023. Neonatal and long-term survival of the general population have improved markedly, as illustrated in **Figure 3** for individuals born in 2023, although the latter data are based on estimates.

**Figure 4.**
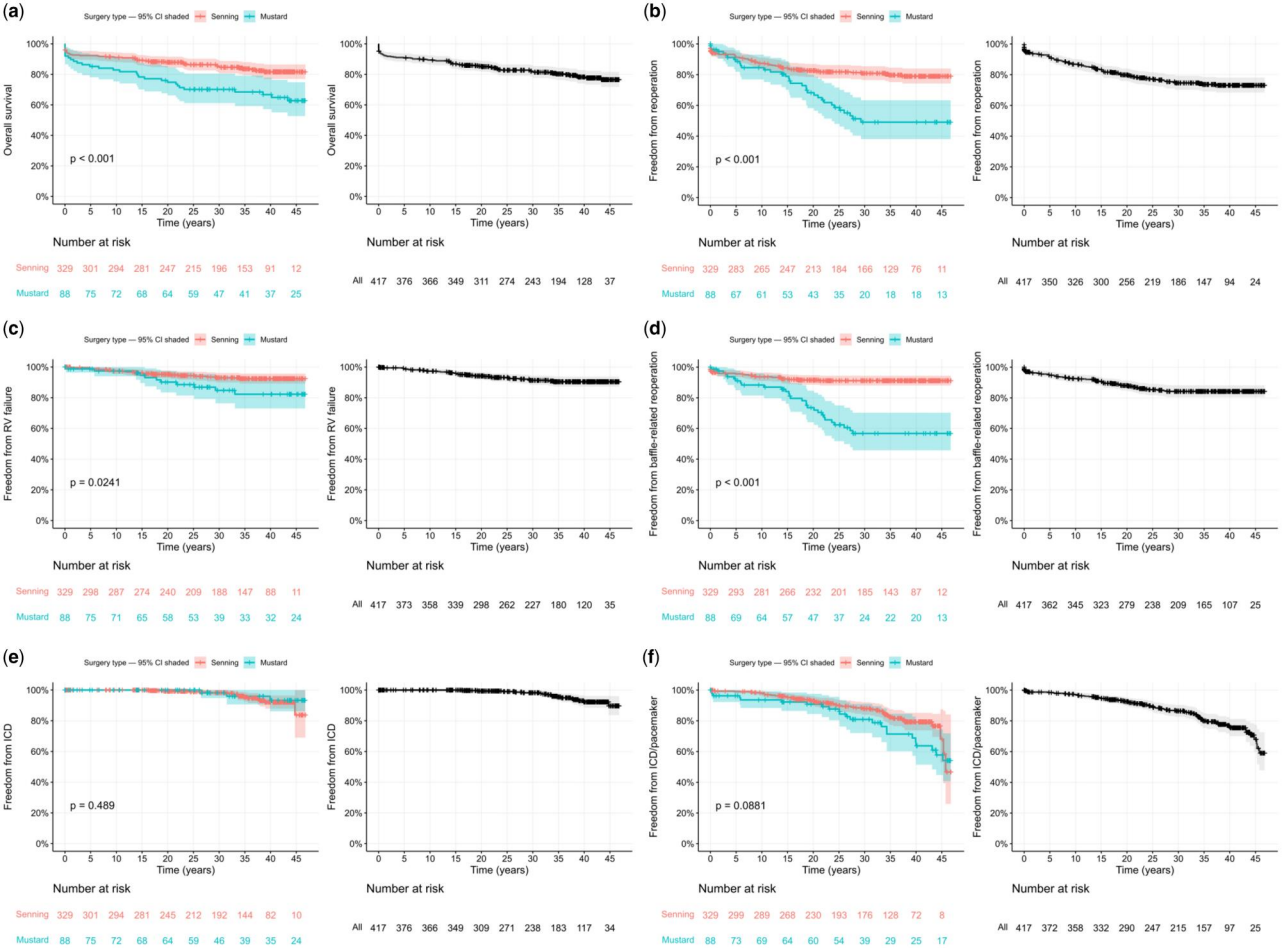
Survival and Freedom From Major Adverse Events After Atrial Switch Repair by Surgical Technique (Colour) and in the Overall Cohort (Black). (A) Overall survival by surgery type (colour) and for the complete cohort (black), including hospital mortality. (B) Freedom from reoperation by surgery type (colour) and for the complete cohort (black), including hospital mortality. (C) Freedom from reoperation for right ventricular failure by surgery type (colour) and for the complete cohort (black). (D) Freedom from baffle-related reoperations by surgery type (colour) and for the complete cohort (black). (E) ICD-free survival by surgery type (colour) and for the complete cohort (black). (F) Freedom from ICD and/or pacemaker implantation by surgery type (colour) and for the complete cohort (black).

There was significant inferiority of the Mustard operation regarding long-term survival when compared to the Senning operation (**Figure 4**).

After the year 20 of observation, the slopes of the survival curves after Senning and Mustard were similar, with most of the increased mortality after Mustard observed within the first 20 years of observation. The divergence of both survival curves was mostly based on a change in slope of the Mustard survival function, with the Senning survival function presenting a relatively similar slope over 40 years (**Figure 4**).

### Incidence of reoperations

Cardiac reoperations were performed in *n* = 95 patients. In *n* = 74 patients, one reoperation was necessary. In *n* = 15 patients, 2 reoperations were performed and *n* = 6 patients underwent 3 or more reoperations. The first reoperation was performed at a median time interval of 8.2 years (range: 0-37 years) after the atrial switch operation, and median patient age at the time of the first reoperation was 8.6 years (range: 0-38 years). Freedom from reoperation at 40 years was 73.1% (95% CI, 0.684-0.780) with *n* = 94 patients at risk (**Figure 4**). Patients with a ventricular septal defect (VSD) were more likely to be treated with a Mustard procedure (22% vs 12% for patch closure, 14% vs 8% for direct VSD closure), which in turn was linked with a higher incidence of reoperations. For other differences between groups, see **Table 1**.

**Table 1.** Patient Characteristics and Perioperative Variables

Characteristics	Missing	Overall group (*n* = 417)	Mustard (*n* = 88)	Senning (*n* = 329)	*P*
Operation before 1983, *n* (%)	0	213 (51.1)	88 (100)	125 (38.0)	<.001
Mean follow-up time, years (SD)	22	29.7 (14.2)	30.2 (16.8)	29.6 (13.4)	.754
Gender, male, *n* (%)	0	293 (70.3)	65 (73.9)	228 (69.3)	.434
Median age at atrial switch, months (IQR)	0	7.9 (2.2–17.2)	22.1 (12.5–46.8)	5.7 (1.7–14.0)	.004
Mean weight at atrial switch, kg (SD)	2	7.5 (4.2)	11.3 (5.3)	6.5 (3.1)	<.001
**Morphology**					
VSD present, *n* (%)	0	130 (31.2)	37 (42.0)	93 (28.3)	.019
Small	0	36 (8.6)	6 (6.8)	30 (9.1)	.669
Moderate or large	0	94 (22.5)	31 (35.2)	63 (19.1)	.002
LVOTO, *n* (%)	0	50 (12.0)	13 (14.8)	37 (11.2)	.360
LV/RV pressure ratio, (SD)	25	0.69 (0.29)	0.73 (0.36)	0.67 (0.27)	.190
Mean aortic oxygen saturation, (SD)	40	64.1 (14.8)	61.0 (10.9)	65.0 (15.6)	.008
**Palliative operation**					
Atrial septostomy, *n* (%)	0	26 (6.2)	21 (23.9)	5 (1.5)	<.001
Banding of pulmonary artery, *n* (%)	0	26 (6.2)	18 (20.5)	8 (2.4)	<.001
Shunt procedure, *n* (%)	0	11 (2.6)	5 (5.7)	6 (1.8)	.059
Brock’s procedure, *n* (%)	0	1 (0.2)	0	1 (0.3)	1.000
Balloon atrial septostomy, *n* (%)	0	382 (91.6)	75 (85.2)	307 (93.3)	.013
CoA repair, *n* (%)	0	10 (2.4)	5 (5.7)	5 (1.5)	.039
Ligation of patent arterial duct, *n* (%)	0	17 (4.1)	8 (9.1)	9 (2.7)	.013
**Surgical**					
Mean cardiopulmonary bypass time, min (SD)	7	105.7 (33.7)	105.3 (33.7)	105.8 (33.7)	.907
Mean ischaemic time, min (SD)	7	56.1 (22.4)	40.4 (28.2)	60.2 (18.6)	<.001
Total circulatory arrest, *n* (%)	7	330 (79.1)	48 (54.5)	282 (85.7)	<.001
VSD closure, *n* (%)	0	94 (22.5)	31 (35.2)	63 (19.1)	.002
VSD closure with direct suture	0	37 (8.9)	12 (13.6)	25 (7.6)	.091
VSD closure with patch	0	57 (13.7)	19 (21.6)	38 (11.6)	.022
Left ventricular outflow tract procedure, *n* (%)	0	50 (12.0)	13 (14.8)	37 (11.2)	.360

SD: Standard deviation; IQR: interquartile range; VSD: ventricular septal defect; LVOTO: left ventricular outflow tract obstruction; LV: left ventricle; RV: right ventricle; CoA: Coarctation of Aorta.

### Indication for reoperations

There were 3 main categories of indications for reoperation. The most frequent indication was a baffle-related complication (*n* = 70 procedures in *n* = 56 patients). Into the second most frequent category of reoperations performed for a pathology related to systemic ventricular failure fell *n* = 31 patients who underwent *n* = 40 surgeries in total. Reoperations for right ventricular failure included PAB (*n* = 11 procedures performed on *n* = 9 Senning and *n* = 2 Mustard patients), conversion to arterial switch operation (*n* = 5 Senning patients), tricuspid valve repair or replacement (*n* = 25 procedures performed on *n* = 12 Senning and *n* = 9 Mustard patients), ventricular assist device implantation (*n* = 1 Senning patient), and transplantation (*n* = 1 Mustard patient). In addition, *n* = 18 patients underwent *n* = 19 reoperations for a left ventricular outflow tract obstruction, where one patient had 2 reoperations.

Freedom from reoperation for right ventricular failure at 40 years was 90.5% (95% CI, 87.2%-93.8%) with *n* = 120 patients at risk (**Figure 4**). Twelve patients underwent tricuspid valve repair where 3 patients had these reoperations twice. Freedom from reoperation for baffle complications at 40 years was 84.2% (95% CI, 80.5%–88.2%) with *n* = 107 patients at risk (**Figure 4**). Freedom from reoperation for left ventricular outflow tract obstruction at 40 years was 94.7% (95% CI, 92.3%–97.2%) with *n* = 123 patients at risk.

Over the first 15 years of observation, the slopes of Kaplan-Meier curves for freedom from any type of reoperation were comparable and the inferiority of the Mustard manifested at 15-30 years of observation.

### Rhythm disturbances

Electrocardiographic data at final follow-up were available for 75% of survivors (*n* = 335). Among patients with available data (*n* = 254), 68.9% remained in sinus rhythm, 18.1% exhibited atrial or junctional rhythm with an adequate chronotropic response, while 13.1% demonstrated an impaired chronotropic response and were managed with a permanent pacemaker already *in situ*. A total of *n* = 125 patients (30%) had a record of hospitalization due to an event of rhythm disturbance. *N* = 16 patients received a cardiopulmonary resuscitation with return of spontaneous circulation (ROSC) on the basis of malignant arrythmia; *n* = 62 patients received a catheter-based electrophysiological examination with ablation, of whom *n* = 25 patients received the examination twice.

### Cardiac device implantation

*N* = 74 patients (18%) received either a permanent pacemaker, implantable cardiac defibrillator (ICD), or combined device. Among these, *n* = 61 patients (82%) received a pacemaker, and *n* = 17 (23%) received an ICD. There were *n* = 4 patients (3%) who received a device with both functions. Freedom from ICD implantation at 40 years was 92.3% (95% CI, 88.5%-96.1%), with *n* = 117 patients at risk (**Figure 4**). Freedom from pacemaker implantation at 40 years was 79.9% (95% CI, 75.0%-85.2%), with *n* = 104 patients under surveillance. Freedom from cardiac device implantation (ICD or pacemaker) at 40 years was 75.4% (95% CI, 70.0%-81.3%), with *n* = 97 patients at risk (**Figure 4**). Both freedom from pacemaker curves for Mustard and Senning decline starting at year 5 of observation, with relatively stable and similar slopes over the decades. In contrast, ICD implantations did not commence before the year 30 of observation, which most likely reflects patterns of clinical practice and technical device development.

## Discussion

This single-centre study demonstrates that survival after the Senning or Mustard operations can extend well beyond 4 decades, with the most pronounced difference in survival rates between the 2 types of atrial switch procedures presenting during the first 20 years after the index operation. Long-term survival up to 4 decades after the Senning procedure is close to that of a general population sample born in the year of commencement of atrial switch operations.

We report extended follow-up of a patient cohort, which has been studied and published in 2006^5^ and in 2008^6^ in the form of an isolated report on the results of the Senning operation. For the mixed Mustard-Senning cohort, the mean follow-up time was extended from 19.1 (SD 6.5) years^5^ to a mean follow-up of 29.7 (SD 14.2) years, and from 7553 to 12.389 cumulative patient-years. The homogeneity of this single-centre patient substrate with central core-lab follow-up allows for fine granularity in analysing follow-up trends over 4 decades. Similar-sized single-centre observational studies have been published among others by Gelatt et al,^7^ Sarkar et al,^8^ Genoni et al,^9^ Moons et al,^10^ Vejlstrup et al,^11^ and Kiener et al.^12^

Multicentre reports and meta-analyses have been reported in recent years, shedding light on some of the central modifiers of long-term outcome after atrial switch. In 2022, Broberg et al^13^ published a multicentre retrospective analysis of 1168 patients after atrial switch operation for d-TGA, reporting a median of 9.2 years of follow-up. This cohort of patients represents a multinational cohort from various countries, with the main finding that adverse events including death, transplantation or need of MCS occur scattered throughout all stages of adult life. Freedom from composite end-point death/transplant/MCS approached 80% at 15 years, a finding to which the herein reported single-centre data compare favourably. A systematic review and meta-analysis published in 2019^14^ included 29 observational studies and 5035 patients after atrial switch, which confirmed Mustard as a procedural factor and the presence of complex forms of TGA as anatomical factors affecting survival.

### Reoperations in general and baffle-specific

After 25 years, a clear trend towards more reoperations of any kind, and more baffle-specific reoperations had been shown by Lange et al^5^ for the Mustard group. Already demonstrating a low freedom from reoperation of only 50% at 25 years, the Mustard group continues to exhibit this unfavourable trend between 25 and 30 years, with the apparent plateau of the Kaplan-Meier curve likely reflecting the limited number of patients remaining under observation beyond 30 years. For the Senning cohort, the most prominent time window regarding reoperations proved to be the first 15 years, with much less steepness and considerably stable rates from 15 to 40 years, with substantial numbers under observation out until 35 years. In contrast, the trend of deterioration for the Mustard group became even more pronounced starting around the year 15 of observation. In conclusion, beyond 15 years of observation, reoperation rates deteriorated for the Mustard while they were stabilizing in the Senning group.

Due to selection bias and surgeons preferring Mustard over Senning for more complex cases, those individuals with a VSD were more frequently treated with Mustard, which must be taken into consideration when interpreting the impact of a VSD on long-term outcome in this specific patient cohort.

### Reoperations for right ventricular dysfunction

Reoperation rates for any procedure performed for right ventricular dysfunction, including tricuspid valve repair or replacement, began to diverge at 15 years, which represents a trend not detectable in prior studies,^5^ with inferiority of the Mustard and very stable curve progression for the Senning operation. Patients after Senning were also reported to be in better functional status up to 15 years after index operation.^10^ The functional status of the cohort had previously been assessed by Lange et al,^5^ who found that it was generally high, with most individuals able to work. However, it gradually declined over time. Notably, functional status did not necessarily correlate with the degree of right ventricular dysfunction.^15^^,^^16^

### Survival

Survival curves for Mustard and Senning procedures diverted within the first 20 years of observation, with consistently inferior results of the Mustard, in addition to considerable perioperative losses in this group.^5^ The curves are in concordance with historical series of Mustard, with almost 10% of early mortality reported,^7^ with a recently published major meta-analysis on 2912 patients, which identified the Mustard as an independent risk factor for mortality with an OR of 2.2 compared to the Senning.^17^ With an OR of 2.9, this finding was also widely confirmed in the analysis reported by Venkatesh et al.^14^ We show a relatively stable survival function between years 25 and 40 for the Mustard group and slightly steeper slope of the curve for the Senning operation in this time window, with very low rates of death for both groups at 25-40 years, exceeding survival described in other reports.^11^ Death rates after Mustard stabilized after 25 years of observation. Deaths reported in historical analyses were mostly sudden, without preceding deterioration of ventricular dysfunction^7^^,^^8^ and with younger age at operation reported as a protective factor by some,^9^ however with limited observation times up to an average of 12-14 years.^8^^,^^9^

The freedom from reoperation curves did not begin to diverge before 15 years of follow-up, indicating that the hazard associated with reoperation is insufficient to account for the observed difference in survival between the 2 cohorts, which became particularly pronounced after 10 years of follow-up.

### Implantable cardiac defibrillator implantation

The occurrence of ICD implantations was only observed after 30 years, with comparable rates in the Mustard and Senning groups. This pattern argues against baffle-related mechanisms as the main cause, since baffle complications in Mustard patients typically manifest within the first 15-20 years.^18–21^ ICD implantations did not commence before year 30 of observation, indicating the former clinical practice pattern rather than the occurrence of malign arrythmias. The first ICD implantation did not occur before 1980 and was performed at Johns Hopkins Hospital. It was approved by the FDA in 1985, approximately 13 years after the first atrial switch operation had been performed at the German Heart Center Munich. In addition, years of technical refinement and prospective trials regarding these devices are likely to have further delayed broad clinical application. The difficult transvenous access following intraatrial baffle repair compared to structurally normal hearts may have also delayed application in this patient population. It is possible that the introduction of ICD and pacemaker devices at around 25 years of observation contributed to improved survival, reflected in the observation of a less steep survival curve in the Mustard group starting at 25 years of observation.

## Conclusion

In conclusion, this study provides updated follow-up data extending into the fourth decade after the atrial switch operation for d-TGA. While hazard of death decreased for the Mustard between years 25 and 40 of observation, after it had been considerably high until the year 25 of observation, the Senning showed consistently stable hazard rates, and the survival rate was slightly inferior to that of a matched general population sample. Beyond year 15, the hazard of right ventricular failure and associated reoperations increased especially for the Mustard operation. Long-term outcomes are not determined exclusively by the type of atrial switch performed but are influenced by prognostic factors such as anatomical substrate,^10^ the degree of tricuspid regurgitation, arrhythmia burden, and baffle-related complications. Very long-term follow-up studies, like the work presented herein, can provide further information on controversial topics such as implantable cardiac defibrillator therapy. The atrial switch operations, especially the Senning operation, are highly reproductive procedures with excellent long-term results and should remain in the armamentarium of today’s congenital cardiac surgeon for rare indications.

### Limitations

Survivorship bias is possible, as patients who died early or lacked follow-up documentation are underrepresented in long-term analyses, which may overestimate survival among late survivors. Temporal and institutional experience bias is very likely, making it difficult to directly compare the long-term survival rates of Mustard and Senning techniques. Outcomes may have improved over time due to advancing surgical expertise and postoperative care rather than being linked to the procedure itself.
